# Cooperative Effects of Cellulose Nanocrystals and Sepiolite When Combined on Ionic Liquid Plasticised Chitosan Materials

**DOI:** 10.3390/polym13040571

**Published:** 2021-02-14

**Authors:** Pei Chen, Fengwei Xie, Fengzai Tang, Tony McNally

**Affiliations:** 1 College of Food Science, South China Agricultural University, Guangzhou 510642, China; peichen@scau.edu.cn; 2 International Institute for Nanocomposites Manufacturing (IINM), WMG, University of Warwick, Coventry CV4 7AL, UK; 3 WMG, University of Warwick, Coventry CV4 7AL, UK; Fengzai.Tang@warwick.ac.uk

**Keywords:** polysaccharide plasticisation, biopolymer thermomechanical processing, biopolymer nanocomposites, nanoclay, cellulose nanocrystals, ionic liquid

## Abstract

Cellulose nanocrystals (CNCs) and/or sepiolite (SPT) were thermomechanically mixed with un-plasticised chitosan and chitosan/carboxymethyl cellulose (CMC) blends plasticised with 1-ethyl-3-methylimidazolium acetate ([C_2_mim][OAc]). Examination of the morphology of these materials indicates that SPT aggregates were reduced when CNCs or [C_2_mim][OAc] were present. Inclusion of CNCs and/or SPT had a greater effect on material properties when the matrices were un-plasticised. Addition of SPT or CNCs altered the crystalline structure of the un-plasticised chitosan matrix. Moreover, a combination of SPT and CNCs was more effective at suppressing re-crystallisation. Nonetheless, the mechanical properties and surface hydrophobicity were more related to CNC/SPT–biopolymer interactions. The un-plasticised bionanocomposites generally showed increased relaxation temperatures, enhanced tensile strength, and reduced surface wettability. For the [C_2_mim][OAc] plasticised matrices, the ionic liquid (IL) dominates the interactions with the biopolymers such that the effect of the nanofillers is diminished. However, for the [C_2_mim][OAc] plasticised chitosan/CMC matrix, CNCs and SPT acted synergistically suppressing re-crystallisation but resulting in increased tensile strength.

## 1. Introduction

Natural biopolymers have attracted tremendous interest in creating new and functional materials due to their renewability, biodegradability, and biocompatibility. Among these polymers is, cellulose composed of d-anhydro-glucopyranose joined together by β-1,4-glycosidic bonds [1]. It is widely available in plants and constitutes the most abundant renewable polymer resource. Regenerated cellulose has found wide application such as in food, biomedicine, agriculture, packaging, water treatment, textiles, and in optical/electrical devices [2]. Chitosan is a linear polysaccharide composed of β-(1,4)-linked *N*-acetyl-d-glucosamine units and is the deacetylated form of chitin, which is generally extracted from marine shell waste streams [3]. Chitosan has been widely studied for application in areas as diverse as food, biomedical treatment, pharmaceutics, cosmetics, water treatment, agriculture, and textiles [3,4,5,6].

Biopolymer-based nanocomposites continue to attract intense research interest since they provide a route to obtaining enhanced properties to meet a range of sustainable application needs. Among various nanofillers, cellulose nanocrystals (CNCs) and nanoclays (e.g., montmorillonite (MMT), and sepiolite (SPT)) are highly interesting as they are also derived from renewable resources, are inherently functional, and have similar hydrophilicity to that of biopolymers. A novel chitosan/CNCs polyelectrolyte–macroion complex with tailorable particle size, shape, and charges was examined for drug delivery applications [7]. Edible chitosan/olive oil/CNCs films were developed with reduced water vapour permeability and water solubility, having potential for food packaging [8]. Layer-by-layer assembled chitosan/CNCs nanocomposite coatings were found to exhibit tuneable oxygen barrier performance, again displaying potential for use in food and drug packaging [9]. Nanoporous membranes based on CNCs and chitosan displayed extremely high removal efficiency for positively charged dyes from water [10]. An epichlorohydrin-crosslinked chitosan/SPT composite absorbent was reported, capable of absorbing both cationic and reactive dyes in water [11]. Bio-inspired films based on chitosan, MMT, and CNCs were prepared using water-evaporation-induced self-assembly, which showed improved mechanical and barrier properties compared with chitosan films without MMT or CNCs [12]. Despite these prior attempts, reports on chitosan-based nanocomposites containing CNCs and/or SPT are limited in number. Moreover, there have been limited reports on chitosan-based nanocomposites prepared by thermomechanical processing which is more industrially relevant and time- and, cost-efficient.

The goal of this work is to understand the effects of inclusion of CNCs and SPT on the structure and properties of chitosan and chitosan/carboxymethyl cellulose (CMC) materials prepared by thermomechanical processing. The carboxylate groups not only render cellulose water-soluble (easier to be processed), but make CMC negatively charged. Thus, polyelectrolyte complexation (PEC) can occur between CMC and the chitosan polycation. Based on PEC, biopolymer materials have been fabricated with superior properties that single biopolymers cannot realise, such as hydrolytic stability [13], barrier properties [14], mechanical properties [15,16,17], and cell adhesiveness [18]. An ionic liquid (IL) was used as a plasticiser for the biopolymer systems described here. ILs that contain a strongly basic, hydrogen-bond-accepting anion (e.g., carboxylates or halides) can disrupt the hydrogen-bonded networks in biopolymers effectively [19]. While much attention has been paid to the processing and plasticisation of biopolymers such as starch, using ILs [19,20,21,22,23,24,25,26,27], limited work has been reported on chitosan-based materials especially polyelectrolyte-complexed materials plasticised by ILs. 

While it is widely accepted that for composites of polymers and nanomaterials, material properties are largely determined by the level of dispersion of the nanomaterial and polymer crystallinity, we propose that interactions between the biopolymer, plasticiser, and nanofillers play a more dominant role in determining material properties here. We also propose that there is an interplay between CNCs and SPT, which determines the morphology, structure, and properties of these materials. This work could enrich our understanding of the formulation–structure–property relationships of multiphasic biopolymer systems.

## 2. Materials and Methods

### 2.1. Materials

Chitosan (poly(β-(1,4)-d-glucosamine), with a degree of deacetylation of >90%, a viscosity of about 100 mPa·s (i.e., 1% solution in 1% acetic acid at 25 °C), and a weight-average molecular mass of about 150k g·mol^−1^, was purchased from Shanghai Ryon Biological Technology Co., Ltd. (Shanghai, China). CMC sodium, with a degree of substitution (DS) of 0.7, a weight-average molecular mass of 90k g·mol^−1^, and a viscosity of 50–100 mPa·s (Brookfield, Toronto, ON, Canada, 2% solution, at 25 °C), was purchased from Shanghai Macklin Biochemical Co., Ltd. (Shanghai, China). The chitosan [28] and the CMC were characterised previously [13]. CNCs were supplied by Nanjing XFNANO Materials Tech Co., Ltd. (Nanjing, China); 1-ethyl-3-methylimidazolium acetate ([C_2_mim][OAc]) (≥95.0%) and SPT by Sigma-Aldrich Company Ltd. (Dorset, UK); formic acid (98% *w*/*w* AR) and NaBr (pure) were purchased from Scientific Laboratory Supplies Ltd. (Nottingham, UK). Deionised water was used in all experiments.

### 2.2. Sample Preparation

Table 1 shows the formulations and codes of the different samples prepared in this work. The matrix was either chitosan only (represented by the letter “A”) or chitosan/CMC (represented by “B”). The samples were prepared by pre-mixing, thermomechanical kneading at 80 °C for 15 min, hot-pressing at 110 °C for 10 min, and conditioning at 57% relative humidity (RH) for three weeks as described in detail previously [13]. The nano-additives used were SPT (“S”) and/or CNCs (“C”). Some of the samples were plasticised by [C_2_mim][OAc] at 20% (“E2”) of the matrix. The suffix “F” indicates the processed samples were in film form. A-F and B-F (without plasticiser or nano-additives) [13] and AE2-F and BE2-F (plasticised by [C_2_mim][OAc] at 20% but without nano-additives) [29], prepared in the same way, were reported previously and were compared with throughout the discussion section of this paper.

### 2.3. Characterisation

Scanning electron microscopy (SEM) imaging was performed using a ZEISS SIGMA field-emission gun microscope (Carl Zeiss AG, Oberkochen, Germany) with an acceleration voltage of 6 kV. The biopolymer films were cryo-fractured using liquid nitrogen and the fractured sections were sputter-coated with gold/palladium before imaging. 

Scanning transmission electron microscopy (STEM) was conducted using a Talos F200X transmission electron microscope (Thermo Fisher Scientific, Waltham, MA, USA) at 200 kV to obtain both bright-field (BF) and high-angle annular dark-field (HAADF) images. Ribbons about 60 nm thick were sectioned from epoxy-embedded sample blocks and subsequently transferred onto holey carbon films on 200-mesh copper grids. No liquid was used during preparation to avoid damaging the samples.

Fourier-transform infrared (FTIR) spectra were collected using a Bruker TENSOR 27 FTIR spectrometer (Bruker Corporation, Billerica, MA, USA) with an attenuated total reflection (ATR) accessory with 32 scans for each sample over a range of 4000–500 cm^−1^ at room temperature (RT). 

X-ray diffraction (XRD) analysis was undertaken using a PANalytical Empyrean X-ray diffractometer (Malvern Panalytical Ltd, Malvern, UK) at 40 kV and 40 mA with a Co target (Kα = 1.790307 Å) and a beam slit of 10 mm. The samples were scanned over an angular range (2*θ*) of 6–40° with a step size of 0.0263° and a step rate of 2.16 s/step. 

Thermo-gravimetric analysis (TGA) was undertaken using a Mettler Toledo TGA apparatus (Mettler Toledo, Columbus, OH, USA) over a temperature range of 30–700 °C at 10 K/min under nitrogen. 

Dynamic mechanical thermal analysis (DMTA) was performed using a Tritec 2000 DMA (Triton Technology Ltd, Nottinghamshire, UK) in the dual cantilever mode with a sample length of 5 mm at a displacement of 0.01 mm. Temperature scans were performed from −100 °C to 180 °C at 2 K/min and 1 Hz. The dynamic storage modulus (*E′*), loss modulus (*E″*), and loss tangent (tan *δ* = *E″*/*E′*) were automatically calculated by the software.

Tensile tests were performed using an Instron 3367 universal testing machine (Norwood, MA, USA) with a 1kN load cell at a crosshead speed of 3 mm/min. As the specimens were in the form of thin sheets, specimen extension was measured by grip separation as recommended by ASTM Standard D882. Young’s modulus (*E*), tensile strength (*σ*_t_), and elongation at break (*ε*_b_) were automatically determined using Instron Bluehill 3 software from at least seven replicates for each sample.

Contact angle (*θ*_c_) data were obtained from sessile tests at RT based on Young–Laplace using an Attension Theta Lite instrument (Biolin Scientific, Manchester, UK). 

## 3. Results

### 3.1. Morphology, Molecular Interactions, and Crystalline Structure of the Chitosan-Based Composites

As shown by the SEM images (Figure S1), all the bionanocomposite films had a cohesive morphology. A/S-F, A/CS-F, B/S-F, and B/CS-F showed scattered white dots or even protruding rods, which could be the SPT nanoparticles. In contrast to A/S-F and B/S-F, white dots were less apparent in BE2/S-F and were not observed in AE2/S-F, suggesting [C_2_mim][OAc] assisted the de-aggregation of SPT to some extent. An IL may enter SPT channels and facilitate the de-aggregation of SPT [30]. In AE2/CS-F and BE2/CS-F, the scattered white dots or protruding rods were slightly visible. CMC or CNCs may also interact with the IL, which could interfere with the de-aggregation effect of the IL on SPT.

**Figure 1:**
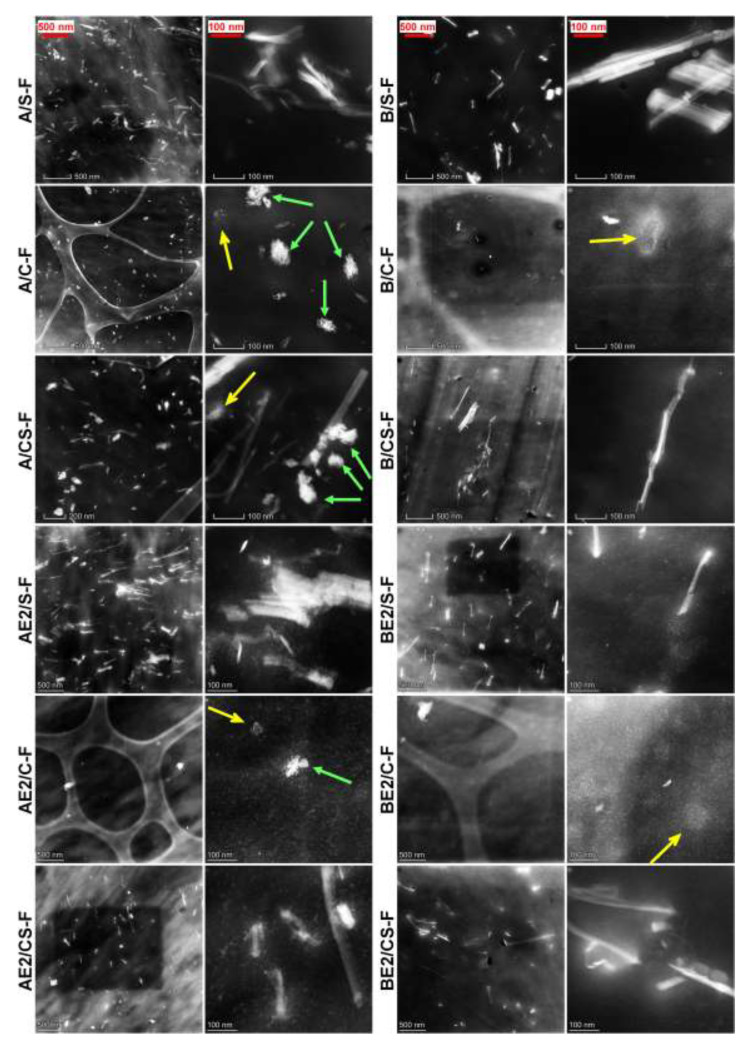
Scanning transmission electron microscopy high-angle annular dark-field (STEM-HAADF) images of the different bionanocomposite films. The green arrows indicate non-dispersed particulate features (chitosan structure); the yellow arrows indicate a “dissolving” feature (likely due to cellulose nanocrystals (CNCs)).

STEM was used to further examine the extent of dispersion of the nanofillers (Figure 1). It was observed that the SPT was well dispersed in the chitosan or chitosan/CMC matrix in all cases, and the length of the SPT needles was in general much shorter than that of the original (Figure S2). It is likely the long needle-like nanoclay was fractured due to the high shear stresses applied during processing. SPT is usually negatively charged in its natural form due to isomorphic substitutions occurring inside the clay platelets, as well as having a hydrophilic character [31,32]. Thus, SPT should have a strong affinity with the chitosan polycation. A/S-F, A/C-F, and A/CS-F also showed some bright dots in HAADF images, which could be derived from unprocessed or re-crystallised chitosan structure (indicated by green arrows). A similar feature was observed previously in the processed chitosan sample (A-F) [13]. Consequently, we speculate that the CNCs under the electron beam appear as fine bright dots which are even less visible under STEM and are associated with “dissolving” features, as highlighted by yellow arrows in A/C-F and A/CS-F (and also B/C-F and BE2/C-F). The CNCs used in this study, obtained by acid hydrolysis, contain negative sulphate half-esters (confirmed from FTIR analysis, see Figure S3) [7,33]. The negative surface charges on CNCs could further enhance its affinity with chitosan. Compared with A/C-F, AE2/C-F exhibited a clearer morphology, suggesting [C_2_mim][OAc] assisted the processing resulting in a more homogenous morphology. Moreover, unlike the A-series, the B-series of samples showed no biopolymer structural features under STEM. This indicates that there are strong interactions between chitosan and CMC (see FTIR results), aiding dispersion during processing and suppressing biopolymer re-crystallisation (see XRD results). 

**Figure 2:**
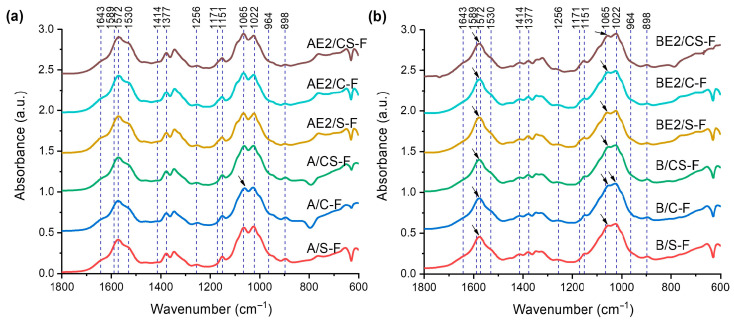
Fourier-transform infrared (FTIR) spectra for the different bionanocomposite films: (**a**) chitosan matrix; and (**b**) chitosan/carboxymethyl cellulose (CMC) matrix. The reference lines indicate characteristic bands for unprocessed CMC (1589, 1414, and 1022 cm^−1^) [13], unprocessed chitosan (1643, 1572, 1530, 1377, 1256, 1151, 1065, 1022, and 898 cm^−1^) [28], [C_2_mim][OAc] (1171 cm^−1^) [29], and SPT (964 cm^−1^) (see Figure S3). The arrows indicate shifts in peak position.

Figure 2 shows the FTIR spectra for the different bionanocomposite films. All the A-series of bionanocomposites displayed quite similar FTIR spectra to that for unprocessed chitosan [28] or A-F [13]. The FTIR spectra for the B-series of bionanocomposites resemble those for the A-series but the peaks were less intense (especially at 1065 cm^−1^ and 1022 cm^−1^). This could indicate strong interactions between chitosan and CMC, manifested by the featureless STEM images and the low degree of crystallinity (see XRD results) obtained for the B-series samples. For the B-series of samples, there was a blue shift of the band originally at 1572 cm^−1^ (N–H bending from amine and amide II) and a red shift of the band at 1065 cm^−1^ (asymmetric C–O–C stretching in the glycosidic linkage) [34,35,36], implying strong molecular interaction between the two polysaccharides. Compared with A-F, A/C-F displayed a red shift of the band originally at 1065 cm^−1^ (asymmetric C–O–C stretching in the glycosidic linkage) [34,35,36]. For B/C-F, this red shift of the band originally at 1065 cm^−1^ was also evident, and there was an additional blue shift of the band at 1022 cm^−1^ (skeletal vibration of C–O stretching) [34,35,36]. These shifts observed for A/C-F and B/C-F may be indicative of strong interactions between the CNCs (a polysaccharide nanofiller) and the polysaccharide matrix. However, for the A- and B-matrices plasticised by 20% [C_2_mim][OAc], such band shifts obtained on inclusion of CNCs were not apparent, suggesting the IL disrupted the interactions between the CNCs and the biopolymers. Furthermore, regardless of matrix type and plasticiser, no significant changes to the bands were observed with the inclusion of SPT, as the interaction of SPT with the biopolymers was weaker than with the CNCs.

**Figure 3:**
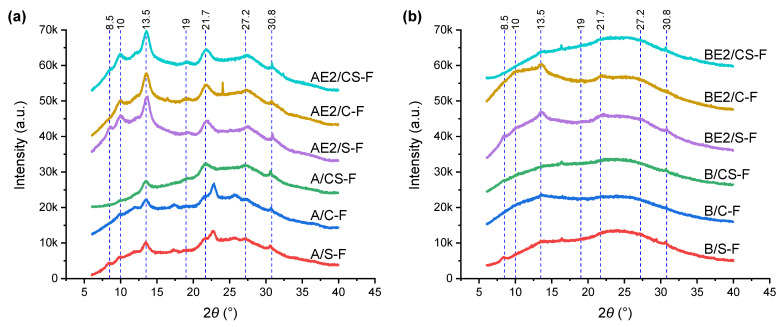
X-ray diffractograms for the different bionanocomposite films: (**a**) chitosan matrix; and (**b**) chitosan/CMC matrix. The reference lines indicate characteristic peaks for sepiolite (SPT) (8.5°) and A-F (the rest) [13].

Figure 3 shows the XRD plots for the different bionanocomposite films. Interestingly, A/S-F and A/C-F displayed very different XRD curves from that for A-F [13] or unprocessed chitosan [28], along with weak peak intensities. It seems that the peak at 21.7° ((100) reflection, 0.48 nm) moved to a higher 2*θ* position (22.8°, *d*-spacing = 0.45 nm) and the peak at 27.2° ((110) reflection, 0.38 nm) moved to a lower 2*θ* position (25.6–25.7°, *d*-spacing = 0.40 nm). Thus, inclusion of CNCs or SPT at a rather low content (0.75 wt%) largely impacted the packing of chitosan chains and suppressed the re-crystallisation of chitosan. In contrast to A/C-F and A/S-F, A/CS-F displayed an XRD curve that matches that for A-F, where the peak intensities were even weaker. In this regard, there may be interaction between CNCs and SPT, which combined results in greater steric hindrance limiting chain movement for re-crystallisation. Compared with AE2-F [29], AE2/S-F, AE2/C-F, and AE2/CS-F exhibited an unchanged XRD pattern, indicating that the inclusion of CNCs and/or SPT did not impact chitosan re-crystallisation.

Compared with B-F which was much less crystalline than A-F [13], B/S-F, B/C-F, and B/CS-F were even more amorphous. Again, the inclusion of both CNCs and SPT was effective at suppressing re-crystallisation of the biopolymers, similar to that observed for A/CS-F. BE2/S-F and BE2/C-F exhibited a similar XRD pattern as that for BE2-F [29]. The IL facilitates biopolymer re-recrystallisation [29]. For BE2/C-F, the peaks at 13.5° and 10° 2*θ* were moderately more intense, suggesting CNCs further assisted the re-crystallisation of the IL-plasticised biopolymers. In contrast, BE2/CS-F was more amorphous, again indicating a cooperative effect of the CNCs and SPT restricting biopolymer re-crystallisation. 

### 3.2. Properties of Chitosan-Based Composites

**Figure 4:**
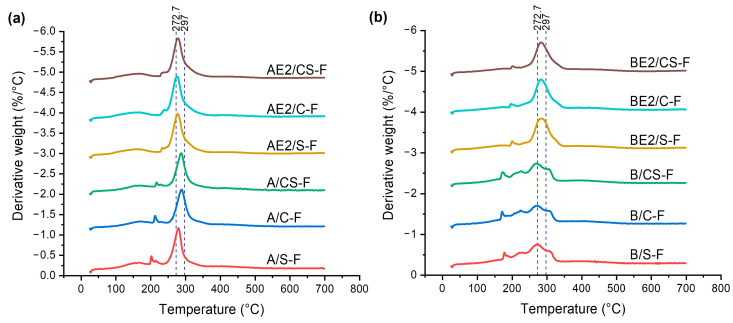
Derivative weight vs. temperature curves measured by thermogravimetric analysis (TGA) for the different bionanocomposite films: (**a**) chitosan matrix; and (**b**) chitosan/CMC matrix. The reference lines indicate the major peak temperatures of B-F (272.7 °C) and A-F (297 °C) [13].

Using TGA, the plots of derivative weight as a function of temperature for the different bionanocomposite films were obtained (Figure 4). Compared with A-F, which had a major decomposition peak temperature (*T*_d_) of 297 °C [13], inclusion of CNCs and/or SPT resulted in reduced thermal stability (*T*_d_ = 280 °C, 289 °C, and 288 °C for A/S-F, A/C-F, and A/CS-F, respectively). CNCs have a *T*_d_ = 290 °C (Figure S4), while SPT is relatively stable with weight loss mainly attributed to free water, zeolitic water, and coordinated water at temperatures up to about 290 °C [28]. Thus, the reduced thermal stability of chitosan on addition of CNCs or SPT is more likely attributed to the lower crystallinity of these samples, as observed from XRD analysis. While the plasticisation of chitosan with [C_2_mim][OAc] (*T*_d_ = 252 °C) reduced the thermal stability of chitosan [29], AE2/S-F, AE2/C-F, and AE2/CS-F displayed slightly higher values of *T*_d_ (278 °C, 275 °C, and 278 °C, respectively) than AE2-F (*T*_d_ = 272 °C). In this regard, the nanofillers may enhance the thermal stability of the plasticised chitosan by restricting the diffusion of pyrolysis products. 

B-F had a *T*_d_ = 273 °C, while the associated peak overlapped with a smaller peak at 306 °C, derived from the polyelectrolyte-complexed structure of biopolymers that were more thermally stable [13]. B/S-F, B/C-F, and B/CS-F displayed a derivative-weight profile similar to that of B-F with no variation in *T*_d_, indicating the lower thermal stability of these samples is due to the CMC. For these samples, the overlapped peak at 306 °C was less sharp, suggesting inclusion of CNCs and/or SPT may have moderately affected the PEC between chitosan and CMC. Compared with BE2-F, BE2/S-F, BE2/C-F, and BE2/CS-F showed no change to the derivative-weight loss profile with *T*_d_ = 283 °C. The plasticisation by [C_2_mim][OAc] led to a more defined peak and the overlapping peak at the higher temperature was significantly diminished, ascribed to the enhanced mixing and interactions between the two biopolymers due to the presence of the IL [29]. Inclusion of CNCs and/or SPT did not alter the role of the IL as the IL dominated the interactions with the biopolymers. 

**Figure 5:**
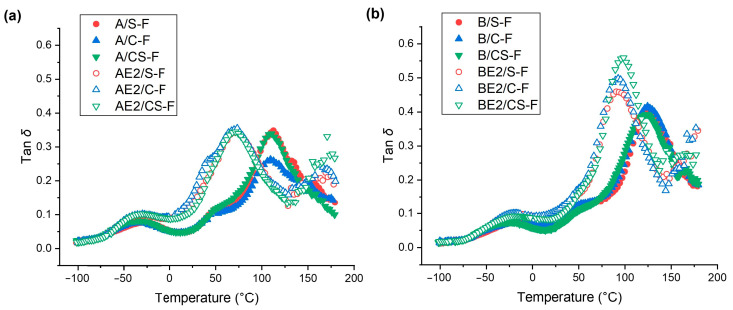
Loss tangent (tan *δ*) *vs.* temperature curves measured by dynamic mechanical thermal analysis (DMTA) for the different bionanocomposite films: (**a**) chitosan matrix; and (**b**) chitosan/CMC matrix.

Figure 5 shows the loss tangent (tan *δ*) curves for the different bionanocomposite films measured by dynamic thermal mechanical analysis (DMTA). A/S-F, A/C-F, and A/CS-F displayed a similar tan *δ* profile to that for A-F [13] with a β-relaxation (the motions of the side chains or lateral groups of chitosan) at sub-zero temperature and the α-transition (glass transition) as shown by a much more prominent peak above room temperature [37,38]. For the un-plasticised A-matrix, inclusion of CNCs and/or SPT resulted in an increase in the peak temperature of the β-relaxation (*T*_β_), from about −47 °C for A-F to about −29 °C for A/S-F, −34 °C for A/C-F, and −35 °C for A/CS-F. This could indicate hydrogen bonding between the nanofillers and the biopolymer side chains or lateral groups, with SPT being more effective (which had additional electrostatic interaction with chitosan and possibly a greater steric hindrance effect). A/S-F, A/C-F, and A/CS-F exhibited an α-transition similar to that for A-F, with the peak temperature of the α-transition (*T*_α_) being about 108–112°C. For the [C_2_mim][OAc] plasticised A-matrix, inclusion of CNCs and/or SPT did not result in changes to both *T*_α_ and *T*_β_. For AE2/S-F, AE2/C-F, and AE2/CS-F, the mobility of either side or main chains should be mainly determined by the plasticiser.

Compared with B-F (*T*_α_ = −43 °C and *T*_β_ = 97 °C) [13], B/S-F, B/C-F, and B/CS-F displayed increases in both *T*_α_ (−19 °C, −18 °C, and −27 °C, respectively) and *T*_β_ (126 °C, 125 °C, and 123 °C, respectively). PEC restricted biopolymer chain mobility and inclusion of CNCs and/or SPT further limited the chain mobility by interacting with the biopolymers. CNCs and SPT combined were less effective at increasing *T*_α_ than either nanofiller alone. In effect, for B/CS-F, which was more amorphous (see XRD results), the mobility of the side chains was less restricted by the nanofillers. For the B-matrix plasticised by [C_2_mim][OAc], *T*_α_ (−23 °C) and *T*_β_ (91 °C) did not vary on inclusion of CNCs or SPT, whereas for BE2/CS-F, *T*_α_ (−18 °C) and *T*_β_ (97 °C) increased slightly. This indicates that, while the IL aided biopolymer chain mobility for the B-matrix, CNCs and SPT combined to reduce the chain mobility of the plasticised biopolymers. 

The stress–strain curves from tensile testing (Figure S5) indicate that all the bionanocomposites were hard and tough materials with different levels of strain hardening obtained. Inclusion of the nanofillers had a greater effect on the mechanical properties when the matrix was un-plasticised. Compared with A-F [13], A/S-F, A/C-F, and A/CS-F were more brittle, whereas B/S-F, B/C-F, and B/CS-F were tougher than B-F. 

**Figure 6:**
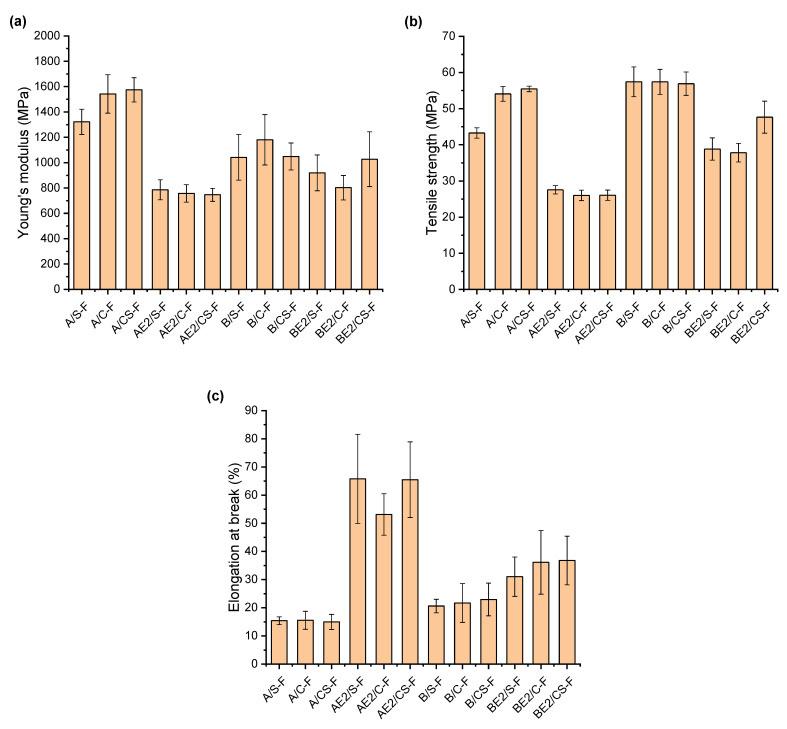
(**a**) Young’s modulus, *E* (**b**) tensile strength, *σ*_t_, and (**c**) elongation at break, *ε*_b_, of the different bionanocomposite films. Error bars represent standard deviations.

From the stress–strain curves, the Young’s modulus (*E*), tensile strength (*σ*_t_), and elongation at break (*ε*_b_) of all the materials were calculated and plotted in Figure 6a–c, respectively. A/S-F, A/C-F, and A/CS-F had lower *ε*_b_ than A-F (22.6 ± 4.6%) [13], indicating increased brittleness. While A/S-F displayed *E* and *σ*_t_ that were not significantly different from those of A-F (*E* = 1260 ± 169 MPa and *σ*_t_ = 46.8 ± 5.6 MPa), A/C-F and A/CS-F exhibited largely increased *E* (1542 ± 152 MPa and 1575 ± 96 MPa, respectively) and *σ*_t_ (54.1 ± 2.0 MPa and 55.5 ± 0.8 MPa, respectively). Thus, we consider the mechanical reinforcement of the un-plasticised A-matrix was mainly provided by the CNCs. As seen from the FTIR results, CNCs interact more strongly with chitosan than SPT. Moreover, the crystalline structure (see XRD results) is not a determinant factor for the mechanical properties of the un-plasticised A-samples. In contrast, for the A-matrix plasticised by [C_2_mim][OAc], inclusion of CNCs and/or SPT did not cause significant changes in mechanical properties of the matrix. In these samples, the hydrogen-bonded network in chitosan was significantly weakened by addition of the IL, and further inclusion of the nanofillers did not impact this plasticisation state, behaviour supported by the FTIR, XRD, and DMTA results.

Compared with B-F (*E* = 1325 ± 176 MPa, *σ*_t_ = 50.5 ± 3.6 MPa, and *ε*_b_ = 10.4 ± 3.4%) [13], inclusion of CNCs and/or SPT led to moderately lower *E* (1042 ± 180 MPa for B/S-F, 1181 ± 199 MPa for B/C-F and 1049 ± 106 MPa for B/CS-F) but similarly higher *σ*_t_ values (e.g., 56.9 ± 3.2 MPa for B/CS-F) and *ε*_b_ (e.g., 22.9 ± 5.8% for B/CS-F). In this regard, the nanofillers may act as crosslinking points increasing the toughness of the un-plasticised B-matrix. PEC results in more effective interfacial stress transfer between the nanofiller (either CNCs or SPT) and the biopolymer matrix. Compared with BE2-F (*E* = 851 ± 181 MPa, *σ*_t_ = 39.1.5 ± 2.6 MPa, and *ε*_b_ = 33.4 ± 8.0%) [13], BE2/S-F, BE2/C-F, and BE2/CS-F displayed similar mechanical properties except that BE2/CS-F had higher *σ*_t_ (47.6 ± 4.4 MPa). As discussed above, the combination of CNCs and SPT provides more hydrogen bonding (i.e., a synergistic effect) to allow more effective stress transfer, thus responsible for the higher *σ*_t_, although BE2/CS was less crystalline. 

Figure S6 shows that the pattern of how inclusion of CNCs and/or SPT influenced the Shore D hardness of the matrices, which generally matches the trends observed for *σ*_t_. These nanofillers were effective at increasing the hardness of the un-plasticised A- or B- matrices whereas, when plasticised by [C_2_mim][OAc], the hardness of the bionanocomposites was similar to the unfilled biopolymer counterparts. While there was increased hydrogen bonding in BE2/CS-F associated with the CNCs and SPT, it was the IL plasticiser that played the major role in determining hardness. 

**Figure 7:**
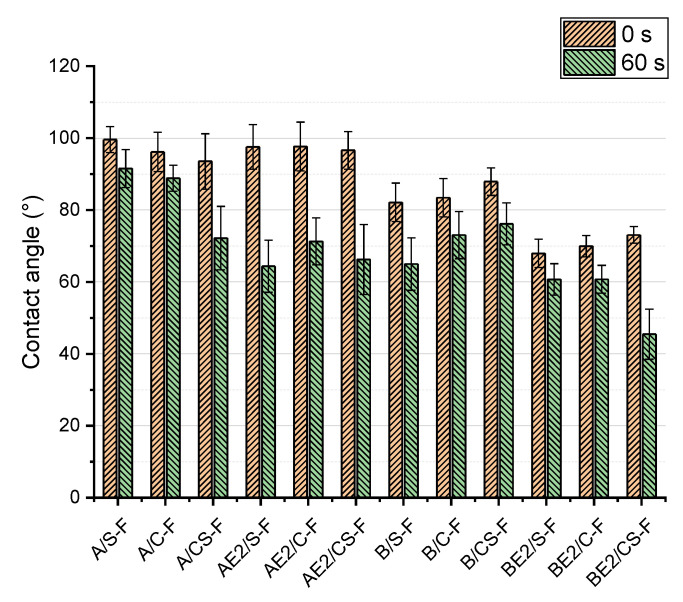
Contact angle values for the different bionanocomposite films at 0 s and 60 s. The error bars represent standard deviations.

Figure 7 shows plots of contact angle (*θ*_c_) values for the different bionanocomposite films. As the contact angle kept changing after the water drop was placed on the biopolymer film surface, contact angles at both 0 s and 60 s (*θ*_c0s_ and *θ*_c60s_, respectively) were recorded. While the surface hydrophilicity/hydrophobicity of a biopolymer material is mainly determined by the free polar groups exposed on the material surface, during wetting, water could destroy hydrogen bonding between biopolymer chains and/or between biopolymer and plasticizer, leading to more free polar groups to bind with water and thus decreasing *θ*_c_ [39]. Compared with A-F (*θ*_c0s_ = 90 ± 5° and *θ*_c60s_ = 68 ± 5°) [13], A/S-F and A/C-F displayed significantly higher *θ*_c60s_ (92 ± 5° and 89 ± 4°, respectively), indicating reduced surface hydrophilicity. In this regard, the strong interaction of CNCs or SPT with chitosan reduces the availability of polar groups (hydroxyl and amine groups) of chitosan to bind with water. However, A/CS-F had *θ*_c0s_ and *θ*_c60s_ similar to those of A-F. In this case, the interaction between the two nanofillers limits their respective interactions with chitosan, leading to unchanged surface hydrophilicity. In contrast, for the [C_2_mim][OAc] plasticised A-matrix, inclusion of CNCs and/or SPT had no significant effect on *θ*_c0s_ and *θ*_c60s_. In this case, the surface hydrophilicity was mainly determined by the interactions between chitosan and [C_2_mim][OAc] and these interactions were on the whole not affected by CNCs or SPT.

For the un-plasticised B-matrix, inclusion of CNCs and/or SPT resulted in increased surface hydrophilicity (*θ*_c0s_ = 82 ± 5° and *θ*_c60s_ = 65 ± 7° for B/S-F, *θ*_c0s_ = 83 ± 5° and *θ*_c60s_ = 73 ± 7° for B/C-F, and *θ*_c0s_ = 88 ± 4° and *θ*_c60s_ = 76 ± 6° for B/CS-F in contrast to *θ*_c0s_ = 71 ± 6° and *θ*_c60s_ = 60 ± 5° for B-F [13]). It is noteworthy that, in this case, CNCs and SPT together also led to reduced surface hydrophilicity, unlike for the case of un-plasticised A-F. As discussed above, PEC results in a more bound structure, the SPT was more widely dispersed shielding the biopolymer polar groups. Moreover, as B/CS-F displayed *θ*_c0s_ and *θ*_c60s_ similar to those of A/CS-F, we consider that the CNCs and SPT counteracted the effect of CMC to increase surface hydrophilicity. Compared with BE2-F (*θ*_c0s_ = 66 ± 6° and *θ*_c60s_ = 47 ± 7°), BE2/S-F and BE2/C-F were less hydrophilic as shown by their higher *θ*_c60s_ (61 ± 4° for both). While BE2-F was more hydrophilic than B-F due to the hydrophilic [C_2_mim][OAc], the CNCs or SPT may have interacted with the IL, thus reducing the overall material hydrophilicity. However, BE2/CS-F displayed *θ*_c0s_ and *θ*_c60s_ similar to those of BE2-F. Again, interaction between CNCs and SPT reduces their respective interactions with the IL or the biopolymers.

## 4. Conclusions

Examination of the morphology of these bionanocomposites indicated that the extent of SPT aggregation could be reduced by inclusion of [C_2_mim][OAc]. Furthermore, the inclusion of CNCs and/or SPT was shown to largely impact the biopolymer crystallinity. Specifically, A/S-F and A/C-F showed a different XRD pattern indicating that SPT or CNCs alter the packing of chitosan chains to form crystals. The combination of SPT and CNCs showed a synergistic effect, more effective at suppressing chitosan re-crystallisation, as seen for A/CS-F, B/CS-F, and BE2/CS-F. However, the material properties were not dependent on crystallinity but more related to the SPT/CNC–biopolymer interactions. While inclusion of CNCs and/or SPT apparently suppressed the crystallinity of the un-plasticised A- and B-matrices, the un-plasticised bionanocomposites generally showed increased relaxation temperatures, enhanced mechanical properties (both *σ*_t_ and *E* for the A-series and only *σ*_t_ for the B-series), and reduced surface wettability. In particular, B/S-F had a *T*_β_ = 126 °C, *θ*_c0s_ = 100 ± 4°, and *θ*_c60s_ = 92 ± 5°; the *σ*_t_ values of A/CS-F and B/CS-F were 55.5 ± 0.8 MPa and 56.9 ± 3.2 MPa, respectively; and *θ*_c0s_ = 88 ± 4° and *θ*_c60s_ = 76 ± 6° for B/CS-F. For the [C_2_mim][OAc] plasticised matrices, the IL dominated the interactions with the biopolymers so that the effect of the nanofillers became weaker. Thus, AE2/S-F, AE2/C-F, and AE2/CS-F had unchanged properties. However, for the IL-plasticised B-matrix, there was a synergistic effect of CNCs and SPT on the biopolymer hydrogen bonding and electrostatic interactions and, thus, mechanical properties (BE2/CS-F had a *σ*_t_ = 47.6 ± 4.4 MPa). Hence, this work demonstrates the importance of tailoring the competing interactions in biopolymer nanocomposite systems for achieving desirable properties.

## Figures and Tables

**Table 1:** Sample codes and compositions (presented as portions by weight).

Sample	Chitosan	CMC	[C_2_mim][OAc]	SPT	CNCs	2M Formic Acid Solution
A/S-F	100	–	–	0.75	–	261
A/C-F	100	–	–	–	0.75	261
A/CS-F	100	–	–	0.325	0.325	261
AE2/S-F	100		20	0.75	–	261
AE2/C-F	100	–	20	–	0.75	261
AE2/CS-F	100		20	0.325	0.325	261
B/S-F	50	50	–	0.75	–	261
B/C-F	50	50	–	–	0.75	261
B/CS-F	50	50	–	0.325	0.325	261
BE2/S-F	50	50	20	0.75	–	261
BE2/C-F	50	50	20	–	0.75	261
BE2/CS-F	50	50	20	0.325	0.325	261

## Data Availability

The data presented in this study are available on request from the corresponding author.

## Supplementary Materials

The following are available online at https://www.mdpi.com/2073-4360/13/4/571/s1, Figure S1: SEM images of the different bionanocomposite films, Figure S2: STEM images of SPT, Figure S3: FTIR spectrum of CNCs and SPT, Figure S4: Derivative weight vs. temperature curve measured by TGA for CNCs, Figure S5: Representative stress–strain curves under tensile testing for different biopolymer composite films, Figure S6: Shore D hardness values of the different biocomposite films.
