# Supplementary material for: Cooperative Effects of Cellulose Nanocrystals and Sepiolite When Combined on Ionic Liquid Plasticised Chitosan Materials
Source: Polymers (Basel). 2021 Feb 14;13(4):571. doi: 10.3390/polym13040571 (PMC7918726; doi:10.3390/polym13040571)
Supplement: Supplementary file 1 [file polymers-13-00571-s001.pdf]

§ Supplementary Material §

Cooperative effects of cellulose nanocrystals and sepiolite when combined on ionic liquid plasticised chitosan materials

Pei Chen [a,b], Fengwei Xie [b,*,†], Fengzai Tang [c], Tony McNally [b,**]

[a] *College of Food Science, South China Agricultural University, Guangzhou, Guangdong 510642, China*

[b] *International Institute for Nanocomposites Manufacturing (IINM), WMG, University of Warwick, Coventry CV4 7AL, United Kingdom*

[c] *WMG, University of Warwick, Coventry CV4 7AL, United Kingdom*

[*] Corresponding author. Email addresses: d.xie.2@warwick.ac.uk, fwhsieh@gmail.com (F. Xie)

[**] Corresponding author. Email address: t.mcnally@warwick.ac.uk (T. McNally)

[†] This author leads the research.

Table of Contents

1 Figures S2

2 Notes to figures S5

References S5

1 Figures

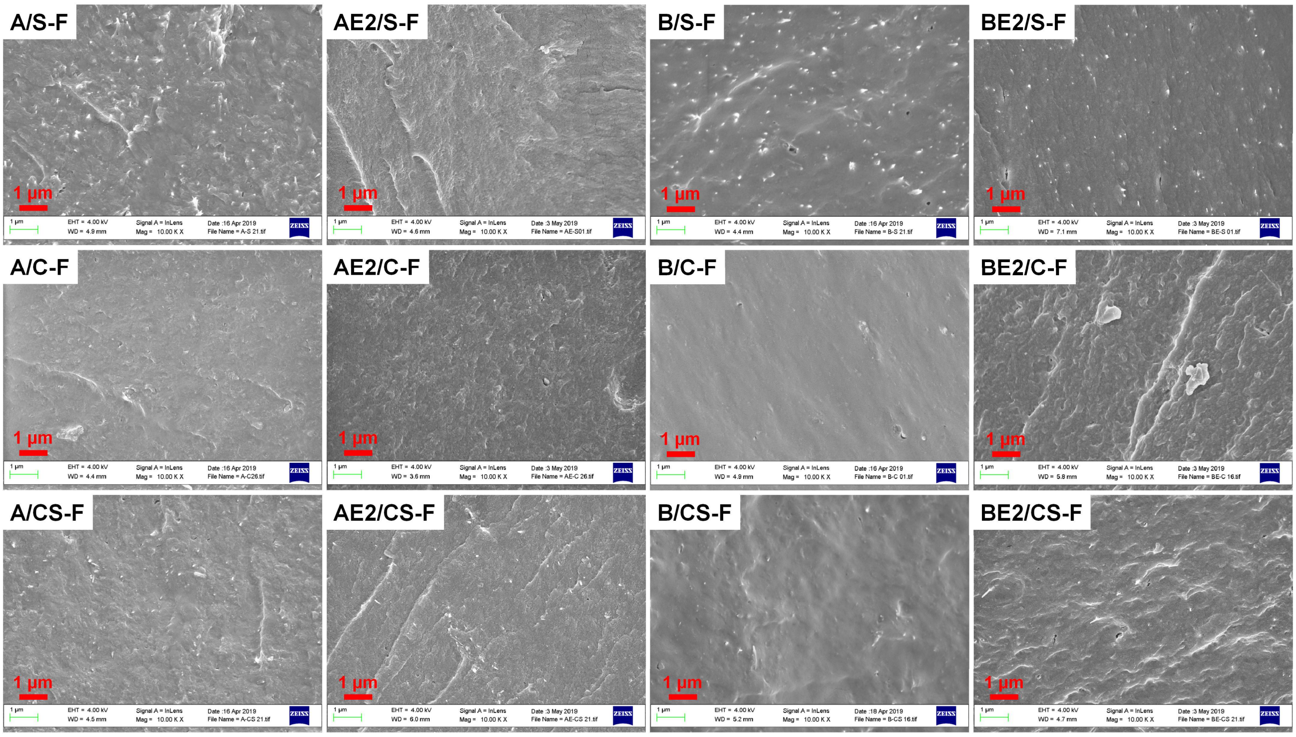

Figure S1. Scanning electron microscopy (SEM) images of the different bionanocomposite films.

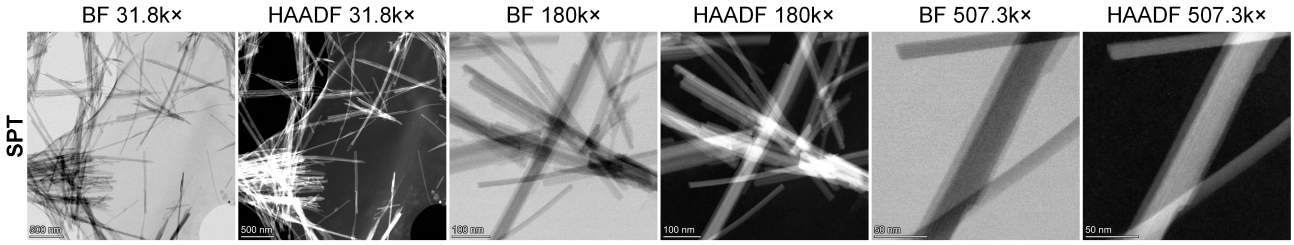

Figure S2. Scanning transmission electron microscopy (STEM) images of sepiolite (SPT). BF, bright field; HAADF, High-angle annular dark-field.

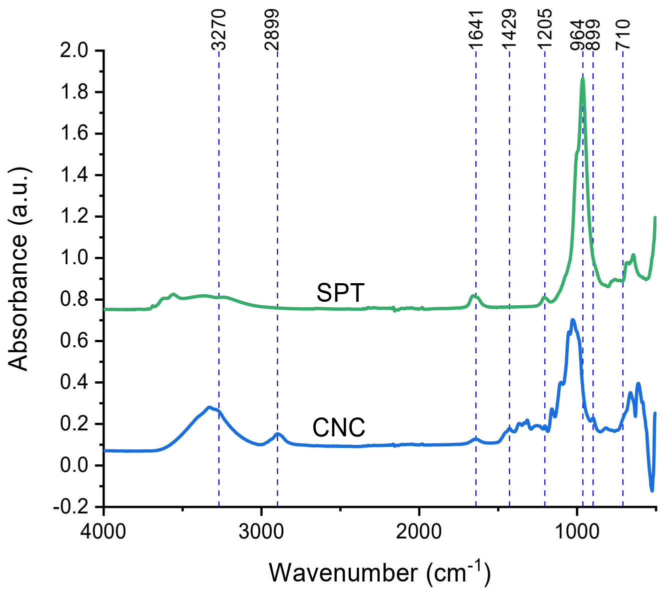

Figure S3. Fourier-transform infrared (FTIR) spectrum of cellulose nanocrystals (CNCs) and sepiolite (SPT).

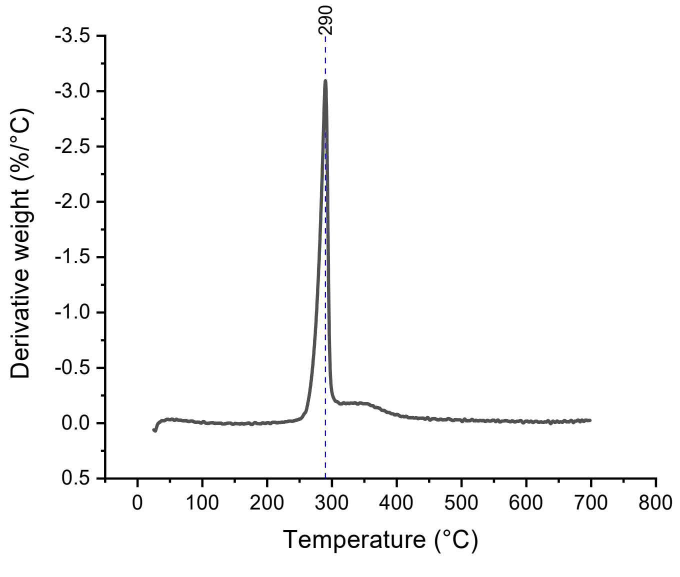

Figure S4. Derivative weight *vs.* temperature curve measured by thermogravimetric analysis (TGA) for cellulose nanocrystals (CNCs).

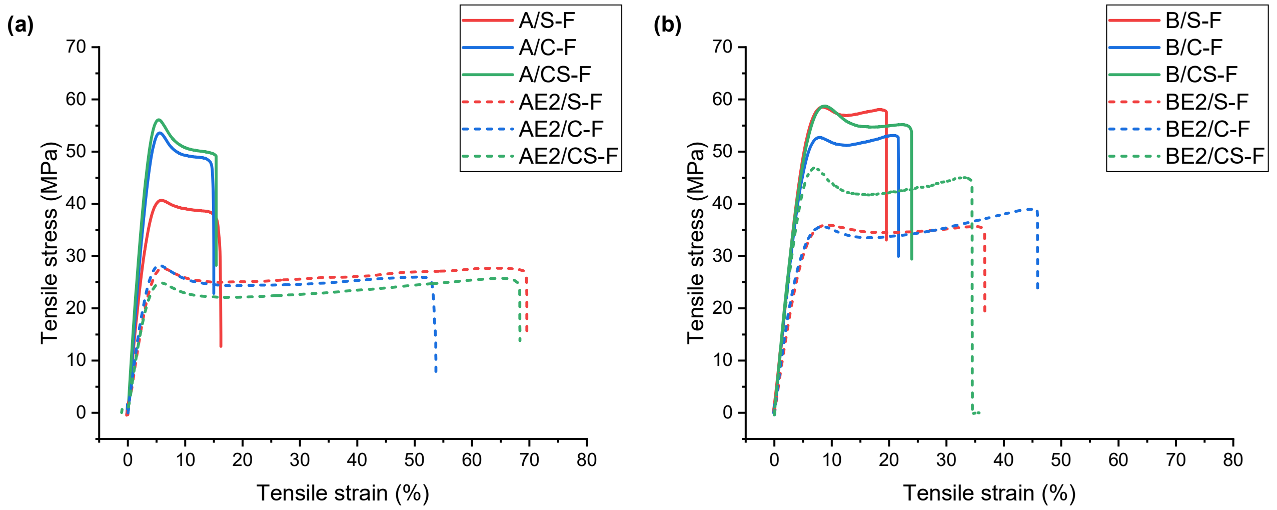

Figure S5. Representative stress–strain curves under tensile testing for different biopolymer composite films: a) chitosan matrix; and b) chitosan/CMC matrix.

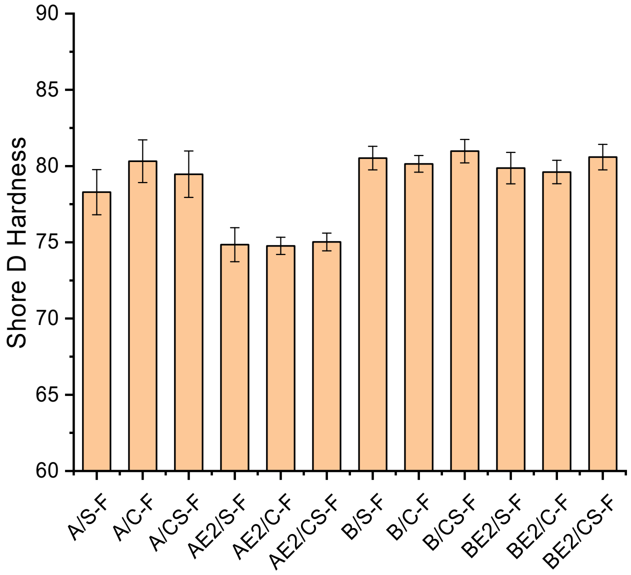

Figure S6. Shore D hardness values of the different biocomposite films.

2 Notes to figures

Figure S3 shows that for cellulose nanocrystals (CNCs), the broad bands in the 3680–3000 cm^{-1} region are due to O─H stretching vibrations and the peak at 2899 cm^{-1} corresponds to C─H stretching vibrations. The 1430 cm^{-1} band is assigned to C6─CH_2 bending [1]. The band at 899 cm^{-1} is attributed to C─O stretching and C─H vibration in cellulose [2]. There is a sulphate peak at 1205 cm^{-1} resulting from the esterification reaction, suggesting this CNCs was obtained by acid hydrolysis. The O─H stretching at 3270 cm^{-1} and the out-of-plane bending at 710 cm^{-1} indicates this CNCs is of the cellulose Iβ type [1]. For sepiolite (SPT), the band at 964 cm^{-1} can be assigned to Si─O stretching [3].

References

1. Lu, P.; Hsieh, Y.-L. Preparation and properties of cellulose nanocrystals: Rods, spheres, and network. *Carbohydr. Polym.* **2010**, *82*, 329-336, doi:10.1016/j.carbpol.2010.04.073.
2. Johar, N.; Ahmad, I.; Dufresne, A. Extraction, preparation and characterization of cellulose fibres and nanocrystals from rice husk. *Ind. Crops Prod.* **2012**, *37*, 93-99, doi:10.1016/j.indcrop.2011.12.016.
3. McKeown, D.A.; Post, J.E.; Etz, E.S. Vibrational analysis of palygorskite and sepiolite. *Clays Clay Miner.* **2002**, *50*, 667-680, doi:10.1346/000986002320679549.
